# *Ephedra alata* Subsp. Alenda as a Novel Source of Bioactive Phytochemicals: Characterization Based on the Mass Spectrometry and Profiling of Antioxidant and Anti-Inflammatory Properties

**DOI:** 10.3390/life13020323

**Published:** 2023-01-23

**Authors:** Afoua Mufti, María del Mar Contreras, Irene Gómez-Cruz, Abdullah Alshamrani, Saber Nahdi, Lamjed Mansour, Salah Alwasel, Abdel Halim Harrath, Nizar Tlili

**Affiliations:** 1 Laboratory of Biotechnology and Biomonitoring of the Environment and Oasis Ecosystems, Faculty of Sciences of Gafsa, Gafsa 2112, Tunisia; 2 Department of Chemical, Environmental and Materials Engineering, Centre for Advanced Studies in Earth Sciences, Energy and Environment (CEACTEMA), Universidad de Jaén, Campus Las Lagunillas, 23071 Jaén, Spain; 3 Department of Zoology, College of Science, King Saud University, Riyadh 11451, Saudi Arabia; 4 Institut Supérieur des Sciences et Technologies de l’Environnement Borj Cédria, Université de Carthage, Hammam chat 2050, Ben Arous, Tunis 1073, Tunisia

**Keywords:** *Ephedra alata*, high-performance liquid chromatography-electrospray ionization-quadrupole-time-of-flight mass spectrometry (HPLC–ESI–QTOF/MS), anti-inflammatory, COX1 and COX2, antioxidants

## Abstract

The aim of the present study was to examine, for the first time, the phytochemical content of *Ephedra alata* pulp extract (EAP) and explore its antioxidant and anti-inflammatory capacities. High-performance liquid chromatography-electrospray ionization-quadrupole-time-of-flight mass spectrometry (HPLC–ESI–QTOF/MS) was used for phytochemical analysis and three in vitro antioxidant assays together with three in vitro anti-inflammatory tests were used for the assessment of biological activity. The HPLC–ESI–QTOF/MS analysis revealed the presence of 42 metabolites, including flavonoids, sphingolipides, fatty acids, ephedrine derivatives, and amino acid derivatives. In vitro findings revealed that EAP has interesting 2,2-diphenyl-1-picrylhydrazyl (DPPH), superoxide, and ferrous ion chelating capacities (IC_50_ values were 0.57 mg/mL, 0.55 mg/mL, and 0.51 mg/mL for DPPH, superoxide radical, and ferrous ion, respectively). Furthermore, EAP showed a noticeable anti-inflammatory ability by inhibiting the two cyclooxygenase isoforms, COX-1 and COX-2 (IC_50_ of 59.1 and 58.8 µg/mL for COX-1 and COX-2, respectively), preventing protein denaturation (IC50 = 0.51 mg/mL), and protecting membrane stabilization (IC50 = 0.53 mg/mL). The results highlighted the use of *Ephedra alata* pulp as a potential source of natural compounds with therapeutic effects for the management of inflammatory disorders.

## 1. Introduction

Medicinal plants have a key role in traditional therapeutic systems. They possess a variety of natural products that can be exploited for the treatment of metabolic disorders [1,2]. It is well known that thousands of plant species and their active molecules are involved in the development of modern drugs [3,4]. Non-steroidal anti-inflammatory drugs (NSAIDs) contribute to the main approaches used in medicine to combat pain, analgesia, and inflammation [5]. In fact, inflammation is a highly dynamic process that is allied to a broad spectrum of human diseases such as cancer, neurodegenerative diseases, cardiovascular diseases, obesity, and diabetes mellitus [6]. Inflammation can be characterized as the first protective response of the body’s immune system, and it is generally accompanied by swelling, redness, pain, heat, and dysfunction [7]. Inflammation and pain can be managed by several approaches, including with NSAIDs. Most NSAIDs act on cyclooxygenases (COX1 and COX2), impairing the release of prostaglandin, blocking the inflammation process, and improving the anti-inflammatory impulses at the action site [8,9]. Indeed, arachidonic acid is converted by the enzymes COX-1 and COX-2 into prostaglandins and thromboxanes. These lipid mediators play central roles in inflammation and pain and regular physiological functions [10].

However, the excessive administration of NSAIDs may induce a variety of negative effects, particularly immune-allergic reactions and cardiovascular problems [11]. Many studies have highlighted the ameliorative role of the bioactive compounds in medicinal plants, not only for inflammation and analgesia but also as antioxidants [12]. Indeed, medicinal plants and their bioactive ingredients can be considered eco-friendly components that can be used in drugs and foods. Among other aspects, it is essential to profile the potential bioactive constituents, toxicity, and pharmacology data to fulfill the requirements of pharmaceutical and novel food applications, e.g., anti-inflammatory potential.

Plants from the *Ephedra* genus, which belongs to the Ephedraceae family, are known by their richness in several biomolecules [13,14]. *Ephedra alata* is a wild species that grows on rocky mountains and in clay regions in arid zones [15]. They are widely used in folk medicine as a potential stimulant and deobstruent and to treat allergies, fever, bronchial asthma, and edema [16]. Recently, many pharmacological studies have investigated the antioxidant, anticancer, and antiviral capacities of *E. alata* [17,18,19]. These potential health benefits are related to their well-known pharmacologically active compounds [20,21]. Recently, Mufti et al. [22] and Noui et al. [23] reported some phytochemical contents of seeds and leaves of *E. alata*. However, to our knowledge, no scientific work has focused on the pharmacological properties of *E. alata* fruits.

For these reasons, and based on the ethnomedicinal uses, the aim of the present study was to determine the free radical-scavenging potency and anti-inflammatory potential of *Ephedra alata* pulp extract (EAP). The metabolite profiling of EAP was also studied using high-performance liquid chromatography (HPLC) coupled with quadrupole-time-of-flight (QTOF) mass spectrometry (MS).

## 2. Materials and Methods

### 2.1. Chemicals and Drugs

All the solvents, chemicals, and drugs used in the current study were purchased from Sigma Aldrich (St Louis, MO, USA). The solvents used were of analytical grades, with the exception of those used for chromatographic purposes, which were of LC-MS-grade. C18 column: Kinetex core-shell, Phenomenex, Barcelona, Spain. COX-1 and COX-2 were from Sigma-Aldrich.

### 2.2. Plant Material, Collection, and Extraction

*Ephedra alata* fruits were collected in June 2019 from Gabes (Southeast Tunisia) and identified by Pr. Ezzeddine Saadoui, National Institute for Research in Rural Engineering Water and Forests (INRGREF, Tunisia). The samples were registered with the voucher number EA-06-01 at the herbarium of the INRGREF. The pulps of the collected fruits were separated, rinsed with sterilized water, dried at room temperature, and separately ground. The powdered material was macerated in 80% methanol for 48 h [24]. Afterward, the solution was filtered via syringe filters (nylon; 0.45 mm pore size). The obtained filtrate was concentrated at 40 °C using a rotary evaporator. The final yield of *E. alata* (EAP) was 12% and the obtained residue was a dark greenish solid. One part was reserved for the chromatographic study and a second part was kept for the in vitro assay.

### 2.3. Phytochemistry (HPLC-DAD-QTOF-MS Analysis)

The chemical composition of EAP was studied using high-performance liquid chromatography (HPLC) (Agilent 1200) (Agilent Technologies, Waldbron, Germany) coupled with quadrupole-time-of-flight (QTOF)-MS and MS/MS (Agilent 6530B Accurate Mass Q-TOF), according to Contreras et al. [25]. An electrospray ionization source was used as the interface and a positive ionization mode was used. Phenolic compounds were separated at 0.35 mL/min using two solvents: solvent A contained Milli-Q^®^ water and formic acid (0.1%, *v/v*) and solvent B contained acetonitrile and formic acid (0.1%, *v/v*). The separation was made using a C18 column (2.1 × 50 mm, 2.7 μm) and a linear gradient of solvent B in A was applicated [26]. The injection volume was 10 μL.

The auto-MS mode was applied and the spectra were acquired over an *m/z* range of 60–1200 Da. The mass correction was performed with a continuous infusion of trifluoroacetic acid ammonium salt (*m/z* 112.9856) and hexakis 1H,1H,3H–tetrafluoropropoxy phosphazine (*m/z* 1033.9881) (Agilent Technologies). MassHunter Qualitative Analysis B.06.00 (Agilent Technologies) was applied for data processing to generate the molecular formula and measure the error, isotopic pattern, and mass score.

### 2.4. The Antioxidant Properties: In Vitro Study

#### 2.4.1. Scavenging Ability toward DPPH

A total of 500 μL of different concentrations of EAP (0.2–1 mg/mL) was added to a mixture of 125 μL of DPPH (0.2 mM) and 375 μL of deionized water. The obtained solutions were placed in the dark for approximately 60 min. Ascorbic acid was used as a positive control. The absorbance was measured at 517 nm [27]. The following formula was used for the calculation of the results:

Inhibition (%) = ((1 − Absorbance of sample)/Absorbance of control)) × 100

The control tube contained all reagents except the samples.

#### 2.4.2. Superoxide Radical Scavenging Assay

The mixture obtained by adding 10 μL of EAP at different concentrations (0.2–1 mg/mL) to 500 μL Tris–HCl buffer (50 mM, pH = 8.2) was placed for 20 min at room temperature. After that, 0.2 mL of pyrogallol (3 mM) was added to the mixture. After 4 min at 25 °C, the absorbance was measured at 325 nm [28]. The positive reference was ascorbic acid. The following formula was used to estimate the scavenging activity:

Inhibition (%) = ((1 − Absorbance of sample)/Absorbance of control)) × 100

#### 2.4.3. Ferrous Ion Chelating Assay

The chelating capacity of EAP on ferrous ions was assessed using the method described by Chew et al. [29]. A total of 100 µL of FeSO_4_ (2 mM) was mixed with 1 mL of EAP at various concentrations (0.2–1 mg/mL). After incubation at 25 °C for 5 min, the solutions were mixed with 0.2 mL of ferrozine solution (5 mM) and kept at 25 °C for 10 min. The positive control was ascorbic acid. The absorbance was read at 562 nm. The Fe^2+^ chelating capacity was estimated using the following formula:

Fe^2+^ chelating rate (%) = ((1 − absorbance of sample)/absorbance of control)) × 100.

### 2.5. In-Vitro Anti-Inflammatory Activity

#### 2.5.1. COX-1 and COX-2 Inhibition Assay

The inhibitory activity of EAP toward the cyclooxygenases enzymes (COX-1 and COX-2) was determined as reported by Husseini et al. [30]. Briefly, EAP and positive controls (morphine and indomethacin) were dissolved in DMSO. The enzyme was mixed with 180 µL of a mixture of Tris-HCl buffer (100 mM; pH = 8.05) and hematin (5 mM) and then mixed with 10 µL of the sample or positive control. Then, the mixture was kept for 5 min at 37 °C. To start the reaction, N,N,N,N-Tetramethyl-*p*-phenylenediamine dihydrochloride (TMPD) and 5 µL of arachidonic acid solution dissolved in methanol were added. The absorbance was read at 610 nm after incubation for 1 h. IC_50_ (50% concentration of inhibitory activity), represents the concentration at which a substance exerts half of its maximal inhibitory effect, expressed in mg/mL, and calculated using Graph Pad Prism.

#### 2.5.2. Inhibition of Protein Denaturation

The mixture consisted of 2 mL of EAP (20–1000 µg/mL), 2.5 mL of Tris buffer (pH 6.4), and 0.5 mL of bovine serum albumin (1%, *w/v*) [31]. In a control tube, the positive standard and the sample were replaced with distilled water. After 10 min of incubation at 36 °C, the mixtures were heated for 6 min at 70 °C. The absorbance was measured at 660 nm. The following formula was used to estimate the % of inhibition:

Inhibition (%) = ((1 − Absorbance of sample)/Absorbance of control) × 100

#### 2.5.3. Ethical Clearance

Ethical permission and agreement for the conducive experimental conditions and use of blood samples from human subjects were provided by the ethical committee of the Department of Biology, University of Gafsa (UG/DB/2009). Informed consent was obtained from all study subjects.

#### 2.5.4. Membrane Stabilization

Blood was collected from healthy donors at the Regional Hospital of Gafsa, Tunisia. The donors did not consume anti-inflammatory drugs for a week or more. A total of 10 mL of blood was first centrifuged at 2500 rpm for 10 min and then washed repeatedly in a saline solution. After that, PBS (pH 7.4) was used to dilute the red blood cells (RBCs) until 10% (*v/v*) suspension was obtained. A control tube contained RBC mixed with a buffer solution. Indomethacin was used as a positive control. Tested samples were prepared at concentrations of 200, 400, 600, 800, and 1000 µg/ mL in PBS. After that, 1 mL of each solution was mixed with 1 mL of RBC and incubated for 20 min at 54 °C. After cooling, the samples were centrifuged at 2100 rpm for 5 min and the absorbance was measured at 560 nm [32]. The percentage of inhibition was estimated according to the following formula:

Inhibition (%) = ((1 − Absorbance of sample)/Absorbance of control)) × 100

### 2.6. Statistical Methods

Data were analyzed using one-way analysis of variance (ANOVA) procedures at a significance level of *p* < 0.05, utilizing Prism 7.01 (GraphPad, San Diego, CA, USA). Separate analyses were conducted for each time point. The results were expressed as the mean ± SD and comparisons between treatment means were made using a Tukey posthoc test.

## 3. Results and Discussion

### 3.1. HPLC-ESI-QTOF-MS Analysis

**Figure 1:**
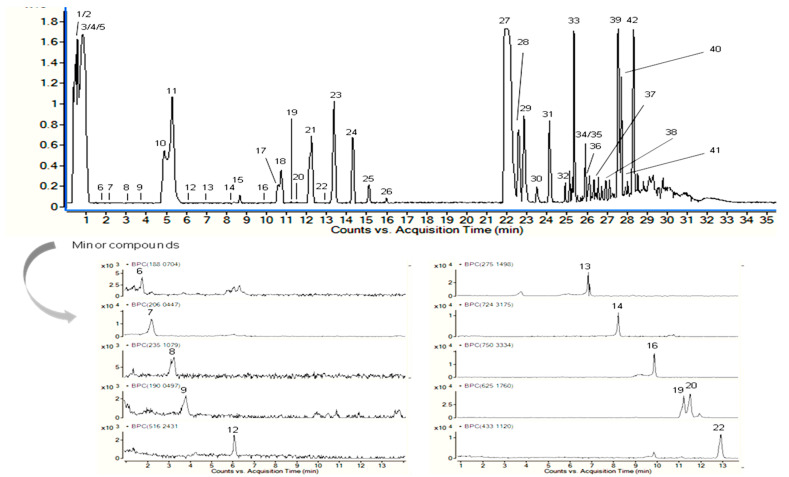
Base peak chromatogram of the *Ephedra alata* pulp (EAP) extract obtained by HPLC-DAD-QTOF-MS analysis.

The compounds found in the EAP were characterized using their molecular formula and fragmentation pattern in addition to information found in the literature and MS spectra databases. Figure 1 shows the base peak chromatograms of the compounds detected by HPLC-QTOF-MS in the positive mode.

Table 1 shows the chemical profile of EAP (42 molecules). Although ephedrine was not detected, four ephedrine derivatives (5, 10, 12, and 14) were found. As a common feature in the MS/MS spectra, they presented a fragment at *m/z* 166.12, which matched the ephedrine molecular formula (Table S1). These compounds have not been reported before, including the sugar derivative of ephedrine (Table 1). Furthermore, kynurenic and hydroxykynurenic acid, which present a quinoline-2-carboxylic acid moiety, and methanoproline were detected in the extract, as has been reported by Caveney et al. [33]. Kaempferol 3-*O*-rhamnoside and isoschaftoside have also been detected in roots and stems of *Ephedra sinica* Stapf [34] and isorhamnetin *O*-glucoside-*O*-rhamnoside has been detected in the fruits of *Ephedra foeminea* Forssk [35]. Sphingolipids, which usually show comparable fragments, including at *m*/z 256.29 (sphingoid base) [36], were also observed. Other observed molecules were leucine (or isoleucine)-hexoside, fatty acids, indoleacrylic acid, phenylalanine-hexoside, and other nitrogen-containing compounds, including the bioactive oleamide. The fragmentation pattern of the last molecule agreed with that found by another study [37]. The other molecules were characterized from *Ephedra* genus for the first time.

### 3.2. Antioxidants Activity

Some medicinal plant compounds inhibit cellular death through their free radical scavenging properties. In the current study, the antioxidant activity of EAP was explored using three methods: Fe^2+^ chelating, superoxide anions, and DPPH assays. The obtained data (Figure S1A) showed that EAP was efficient against DPPH radicals when compared to ascorbic acid (AA). The IC_50_ values were 0.57 mg/mL and 0.54 mg/mL for EAP and AA, respectively. Recent studies have reported that aerial parts and seeds of *E. alata* exhibited important antioxidant potential against DPPH radicals [22,23].

Superoxide, a precursor of several reactive oxygen species, is a toxic radical in cells. The scavenging effects of EAP and AA on the superoxide radical were dose-dependent (Figure S1B). Data from Table 2 show that EAP exhibited an interesting scavenging activity of superoxide radicals (IC_50_ = 0.55 mg/mL) compared to ascorbic acid (IC_50_ = 0.63 mg/mL). The potent antiradical capacity of EAP might be an indication of superoxide anion use by the plant extract [38]. Hamoudi et al. [39] also showed a significant antioxidant capacity using superoxide assay in *Ephedra nebrodensis* extract.

The highest Fe^2+^ chelating activity of EAP (70.6%) was found at 1 mg/mL (Figure S1C). Table 2 shows that EAP presented a similar powerful Fe^2^+-chelator (IC_50_ = 0.51 mg/mL) when compared to the positive standard (IC_50_ = 0.46 mg/mL). The obtained results suggested that the iron-chelating capacity of EAP may be attributed to the presence of various antioxidants that are able to chelate metal ions [39].

The present findings show that *E. alata* pulp could be a source of natural antioxidants against free radicals. In fact, the differences between *E. alata* and other species might be attributed to their biomolecule content [40]. Furthermore, the synergetic effect of bioactive metabolites in the extract may control the antioxidant effect of medicinal species [41]. Carocho and Ferreira [42] reported that the mechanisms involved in the assays used to estimate antioxidant properties are varied and that plants extracts can have different molecules with specific capacities that participate in antioxidant effects, suppressing the formation of reactive oxygen species by inhibiting antioxidant enzymes or chelating trace metals implicated in free radical release, thus forming stable products that do not start or propagate radical production.

### 3.3. In Vitro Anti-Inflammatory Activity

The anti-inflammatory activity of EAP was estimated using three based assays: protein denaturation inhibition, membrane stabilization, and cyclooxygenase inhibition.

**Figure 2:**
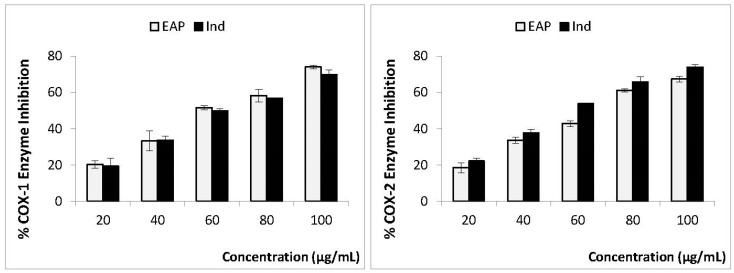
Percentage enzyme inhibition and IC_50_ values of EAP and indomethacin against COX-1 and COX-2 enzymes. *n* = 3, mean ± SD values. IC_50_ (50% concentration of inhibitory activity).

Two isoforms, cyclooxygenase-1 and cyclooxygenase-2, are well-known contributors to the inflammation process [43]. In order to evaluate the cyclooxygenase inhibition profiles of EAP, in vitro COX-1 and COX-2 inhibition assays were carried out using indomethacin as a reference drug. The data obtained revealed that both EAP and indomethacin were able to inhibit both COX-1 and COX-2 at low concentrations (Figure 2). The inhibition effect of EAP on these two cyclooxygenases was dose-dependent (concentrations from 20 to 100 µg/mL), and the highest inhibition capacities of COX-1 (74%) and COX-2 (67%) were detected at 100 µg/mL. It is also interesting to note that the anti-cyclooxygenase propriety of EAP (IC_50_ of 59.1 and 58.8 µg/mL for COX-1 and COX-2, respectively) was similar to that obtained with indomethacin (IC_50_ of 61.8 and 56.7 µg/mL for COX-1 and COX-2, respectively). To our knowledge, this study is the first to evaluate the in vitro anti-cyclooxygenase capacity of *E. alata* pulp extract and is in agreement with previous studies reporting that various medicinal plants are able to inhibit COX1 and COX2 enzymes [44,45].

**Figure 3:**
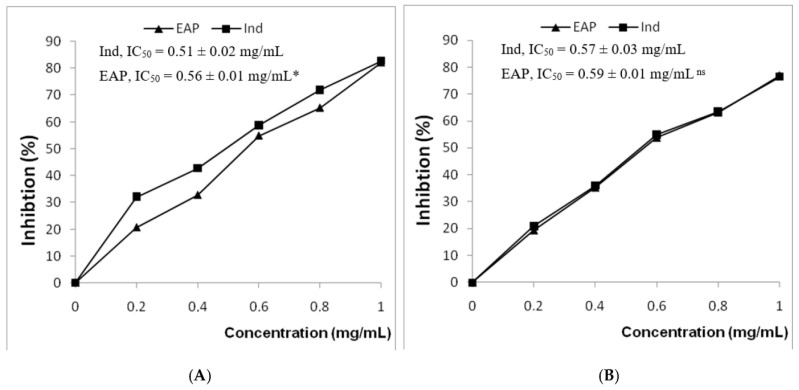
Effect of EAP extracts on heat-induced albumin denaturation (**A**) and hemolysis of erythrocyte membrane (**B**). The data are presented as mean values (*n* = 3). EAP: *Ephedra alata* pulp extract. Ind: indomethacin. IC_50_ (50% concentration of inhibitory activity) values express the concentration for each sample in mg/mL. * *p* < 0.05 significant differences compared to indomethacin. ns: not significant.

Protein denaturation has been correlated with the formation of inflammatory disorders. Therefore, the ability of a substance to prevent protein denaturation is an important step in the development of potential anti-inflammatory medicines [46]. In the present study, the capacity of EAP to block the thermal denaturation of albumin was explored. As shown in Figure 3, EAP and indomethacin inhibited heat-induced albumin denaturation in a dose-dependent manner. The inhibition efficiencies of EAP and the reference drug at 1 mg/mL were 82.2% and 82.7%, respectively. Table 2 shows that the anti-inflammatory capacity of EAP (IC_50_ of 0.51 mg/mL) was similar to that of indomethacin (IC_50_ of 0.56 mg/mL). A recent study corroborating these findings demonstrated that extracts of *Ephedra nebrodensis* could protect protein against denaturation [39]. In the same context, various medicinal plant extracts have been assessed for their ability to inhibit protein denaturation [47,48,49]. Furthermore, it has been suggested that the inhibition of BSA denaturation was responsible for the anti-inflammatory effects of a variety of NSAIDs, such as diclofenac sodium, salicylic acid, indomethacin, and flufenamic acid [50].

The membrane stabilization assay was used to confirm the anti-inflammatory capacity of EAP. Indeed, previous studies have reported that thermal stimuli induce the break of the erythrocyte membrane [51]. Figure 3 shows that EAP and standard indomethacin were able to protect red blood cells (RBC) from heat-induced erythrocyte hemolysis. EAP displayed remarkable anti-hemolytic activities, with an IC_50_ value of 0.57 mg/mL, in a manner similar to indomethacin (IC_50_ = 0.59 mg/mL). The maximum inhibitions of hemolysis of EAP and indomethacin (76.9% and 76.3%) were observed at a concentration of 1 mg/mL. Several studies have supported the ability of plant extracts to stabilize the RBC membrane in a hypotonic solution and inhibit hemolysis [49,52]. In fact, according to Morales León et al. [53], the membrane stabilizer effect could be attributed to the presence of biomolecules in extracts which posses anti-inflammatory properties. Biomolecules and their synergistic have exhibited significant protection of the cell membrane from harmful drug. These compounds were able to interfere with the liberation of phospholipases that activate the production of inflammatory mediators [54].

Furthermore, it has been reported that the deformability and volume of erythrocytes are directly related to the intracellular level of calcium [55]. The ability of the molecules to alter the level of calcium was a probable explanation for the stabilizing activity of the extract. The in vitro anti-inflammatory activity of leaf extracts of *Basella alba*. displayed membrane stabilization effects by inhibiting hypotonicity-induced lysis of the erythrocyte membrane [56].

The present results were concomitant with the findings of Bourgou et al. [18], who investigated the in vitro anti-inflammatory capacity of the aerial parts of two *Ephedra* species from Tunisia (*E. fragilis* Desf and *E. alata*). All these results suggest that EAP can be used as a natural therapeutic against some inflammatory disorders.

It should be noted that inflammation is a very complex process involving the sequential activation of signaling molecules and proinflammatory mediators such as prostaglandins, leukotrienes, and oxygen free radicals [57]. Cyclooxygenase (COX) is the major enzyme responsible for the conversion of arachidonic acid (produced as a result of cell membrane damage) into prostaglandins. Prostaglandins, specifically prostaglandin E2, increase the sensitivity of nociceptors to stimuli and are important mediators of pain and other inflammatory symptoms. The interaction assay with enzymes and their possible inhibitors is an interesting step in the development of potential anti-inflammatory medicines [3,58].

The current findings demonstrate an in vitro inhibition capacity of EAP against COX-1 and COX-2, supporting the use of *Ephedra alata* as a potential source of biomolecules that can be administrated as an anti-inflammatory component.

The data of the present study reveal for the first time that EAP exhibits remarkable anti-inflammatory capacities. The ameliorative effect of EAP might be attributed to its phytochemicals metabolites. Previous findings have reported the anti-inflammatory effects of ephedrine [59], kaempferol [60], isorhamnetin [61], isoschaftoside [62], and oleamide [63], which are some of the compounds (or related compounds) that can be detected in *E. alata*. In addition, the detected sphingolipids in EAP can provide effective drug targets against pathological inflammation [64]. The anti-inflammatory properties of kynurenic acid should not be ruled out [65]. Nonetheless, besides the positive benefits of, for example, ephedrine derivatives and kynurenic acid, much research is required due to the “double-edged sword” of this type of molecule [66]. In fact, it has been shown that various bioactive molecules can bind to COX-1 and COX-2 and induce inhibitory effects on the enzymes [52]. Limongelli et al. [67] reported that in COX-1, the space of the selectivity pocket is diminished due to the presence of isoleucine, while in COX-2, the presence of valine enlarges the existing space, offering a more stable binding possibility for molecules inhibitors.

Future studies will be conducted to purify and elucidate the concrete chemical structure, including its stereochemistry and the biological functions of the molecules found in this extract with promising anti-inflammatory potential. Especially relevant is the presence of new ephedrine derivatives that could have a pharmacological role as ephedrine has in current medication [68].

## 4. Conclusions

Overall, the findings obtained herein show that hydromethanol extracts of *E. alata* pulp has interesting DPPH-, superoxide-, and iron-scavenging capacities. EAP showed a higher hemolysis and protein denaturation inhibition activity. This beneficial effect could be mediated by the inhibition of cyclooxygenase 1 and 2, as detected by anti-cyclooxygenase test studies. The HPLC–ESI–QTOF/MS analysis indicated that EAP contains a mixture of beneficial bioactive compounds that exhibit antioxidant and anti-inflammatory abilities. However, the mechanisms involved in the obtained pharmacological properties deserve further study. Additionally, the chemical structure of *E. alata* compounds and their biological actions must be elucidated.

## Figures and Tables

**Table 1:** Compounds tentatively identified in *Ephedra alata* pulp (EAP) extracts. ^a^ Referred to the sum of the peak areas of the compounds.

No.	Proposed Compound	Molecular Formula	RT (min)	Mean Peak Area	Relative % ^a^
**1**	Unknown	C_5_H_13_NO	0.4	6,941,206	3.567 ± 0.013
**2**	Methanoproline	C_6_H_9_NO_2_	0.5	327,766	0.168 ± 0.004
**3**	Leucine/Isoleucine hexoside	C_12_H_23_NO_7_	0.6	9,895,290	5.085 ± 0.158
**4**	Phenylalanine hexoside	C_15_H_21_NO_7_	0.8	32,493,414	16.699 ± 0.309
**5**	Ephedrine derivative 1 (+ hexosyl + deoxyhexosyl)	C_22_H_35_NO_10_	1.1	47,146	0.024 ± 0.004
**6**	Indoleacrylic acid	C_11_H_9_NO_2_	1.7	18,548	0.010 ± 0.001
**7**	Hydroxykynurenic acid	C_10_H_7_NO_4_	2.2	110,717	0.057 ± 0.001
**8**	Unknown	C_12_H_14_N_2_O_3_	3.2	58,993	0.030 ± 0.001
**9**	Kynurenic acid	C_10_H_7_NO_3_	3.7	26,694	0.014 ± 0.003
**10**	Unknown	C_10_H_13_NO_2_	4.9	6,082,634	3.126 ± 0.216
**11**	Unknown	C_10_H_13_NO_2_	5.3	12,797,772	6.577 ± 0.119
**12**	Ephedrine derivative 2	C_24_H_37_NO_11_	6.1	7645	0.004 ± 0.000
**13**	Unknown	C_14_H_18_N_4_O_2_	6.9	160,758	0.083 ± 0.003
**14**	Ephedrine derivative 3	C_35_H_49_NO_15_	8.2	39,773	0.020 ± 0.004
**15**	Isoschaftoside	C_26_H_28_O_14_	8.7	558,228	0.287 ± 0.001
**16**	Ephedrine derivative 4	C_37_H_51_NO_15_	9.9	44,981	0.023 ± 0.002
**17**	Unknown	C_29_H_59_NO_9_	10.7	1,133,485	0.583 ± 0.012
**18**	Unknown	C_29_H_59_NO_9_	10.8	1,919,159	0.986 ± 0.106
**19**	Isorhamnetin *O*-hexoside-*O*-deoxyhexoside 1	C_28_H_32_O_16_	11.2	312,278	0.160 ± 0.010
**20**	Isorhamnetin *O*-hexoside-*O*-deoxyhexoside 2	C_28_H_32_O_16_	11.5	375,236	0.193 ± 0.000
**21**	Unknown (compound 18 + C_6_H_11_NO)	C_35_H_70_N_2_O_10_	12.3	5,044,989	2.593 ± 0.091
**22**	Kaempferol 3-*O*-rhamnoside	C_21_H_20_O_10_	12.9	89,651	0.046 ± 0.000
**23**	Unknown (compound 19 + C_6_H_11_NO)	C_41_H_81_N_3_O_11_	13.4	2,472,859	1.271 ± 0.047
**24**	Unknown (compound 20 + C_6_H_11_NO)	C_47_H_92_N_4_O_12_	14.3	565,680	0.291 ± 0.006
**25**	Unknown (compound 21 + C_6_H_11_NO)	C_53_H_1_0_3_N_5_O_13_	15.1	74,822	0.038±0.000
**26**	Tetradecasphinganine	C_14_H_31_NO_2_	16	675,136	0.347 ± 0.010
**27**	Hexadecasphinganine	C_16_H_35_NO_2_	22	47,041,954	24.176 ± 1.961
**28**	Phytosphingosine	C_18_H_39_NO_3_	22.7	58,02,618	2.982 ± 0.226
**29**	Sphingolipid derivative 1	C_16_H_35_NO_3_	22.8	6,817,998	3.504 ± 0.513
**30**	Sphingolipid derivative 2	C_16_H_33_NO_3_	23.5	627,400	0.322 ± 0.011
**31**	Unknown	C_14_H_31_NO	24.1	8,684,421	4.463 ± 0.566
**32**	Sphingolipid derivative 3	C_18_H_39_NO_3_	25	2,066,875	1.062 ± 0.186
**33**	Unknown	C_16_H_36_NO	25.4	4,771,780	2.452 ± 0.105
**34**	9,10-Dihydroxystearic acid	C_18_H_36_O_4_	25.8	32,234	0.017 ± 0.000
**35**	Deoxysphinganine	C_18_H_39_NO	25.9	403,861	0.208 ± 0.017
**36**	Hydroxyoctadecatrienoic acid	C_18_H_30_O_3_	26.3	1,173,301	0.603 ± 0.025
**37**	Unknown (choline derivative)	C_28_H_47_N_3_O_6_	26.5	80,220	0.041 ± 0.002
**38**	Unknown	C_20_H_37_NO_3_	27	148,727	0.076 ± 0.004
**39**	Unknown	C_17_H_32_N_6_O_2_	27.5	10,892,423	5.598 ± 0.659
**40**	Oleamide	C_18_H_35_NO	27.8	13,817,387	7.101 ± 0.705
**41**	N-Palmitoylsphingosine	C_34_H_67_NO_3_	28	1,601,879	0.823 ± 0.440
**42**	Unknown	C_19_H_36_N_6_O_2_	28.4	8,344,368	4.288 ± 0.626

**Table 2:** In vitro antioxidant activities of EAP evaluated using DPPH, superoxide, and Fe^2+^ chelating assays at different concentrations. * Results are expressed as IC_50_ (mg/mL).

Sample	DPPH Radical Scavenging	Superoxide Radical Scavenging	Fe^2+^ Chelating
*Ephedra alata* pulp	0.57 ± 0.05 ^ns^	0.55 ± 0.01 *	0.51 ± 0.02 *
Ascorbic acid	0.54 ± 0.01	0.63 ± 0.02	0.46 ± 0.01

Ascorbic acid was used as a positive control. Values are means ± SD of three separate experiments. * *p* < 0.05 significant differences compared to ascorbic acid. ^ns^: not significant.

## Data Availability

Not applicable.

## Supplementary Materials

The following supporting information can be downloaded at: https://www.mdpi.com/article/10.3390/life13020323/s1, Figure S1. Antioxidant activities of EAP evaluated using DPPH (A), Superoxide (B) and Fe^2+^ chelating assays (C) in different concentration. Ascorbic Acid was used as a positive control. Values are means ± SD of three separate experiments EAP: Ephedra alata pulp. AA: Ascorbic Acid; Table S1. Characterization of the compounds tentatively identified in Ephedra alata pulp (EAP) extract.
