# Supplementary material for: Ephedra alata Subsp. Alenda as a Novel Source of Bioactive Phytochemicals: Characterization Based on the Mass Spectrometry and Profiling of Antioxidant and Anti-Inflammatory Properties
Source: Life (Basel). 2023 Jan 23;13(2):323. doi: 10.3390/life13020323 (PMC9961366; doi:10.3390/life13020323)
Supplement: Supplementary file 1 [file life-13-00323-s001.zip › life-2069564-supplementary.pdf]

Article

Ephedra alata Subsp. Alenda as a Novel Source of Bioactive Phytochemicals: Characterization Based on the Mass Spectrometry and Profiling of Antioxidant and Anti-Inflammatory Properties

Afoua Mufti [1], María del Mar Contreras [2], Irene Gómez-Cruz [2], Abdullah Alshamrani [3], Saber Nehdi [3], Lamjed Mansour [3], Salah Alwasel [3], Abdel Halim Harrath [3,*] and Nizar Tlili [4]

[1] Laboratory of Biotechnology and Biomonitoring of the Environment and Oasis Ecosystems, Faculty of Sciences of Gafsa, Gafsa 2112, Tunisia
[2] Department of Chemical, Environmental and Materials Engineering, Centre for Advanced Studies in Earth Sciences, Energy and Environment (CEACTEMA), Universidad de Jaén, Campus Las Lagunillas, 23071 Jaén, Spain
[3] Department of Zoology, College of Science, King Saud University, Riyadh 4545, Saudi Arabia
[4] Institut Supérieur des Sciences et Technologies de l'Environnement Borj Cédria, Hammam chat 2050 Ben Arous, Université de Carthage, Tunisia
* Correspondence: hharrath@ksu.edu.sa.; Tel.: +96653586279

Table S1. Characterization of the compounds tentatively identified in *Ephedra alata* pulp (EAP) extract.

No	*m/z* [M+H]+	Score	Error (ppm)	Compound	Main fragments (*m/z*)
1	104,11	99,1	-3,1	Unknown	
2	128,07	81,8	1,9	Methanoproline	82.0659, 55.0554
3	294,15	99	0,8	Leucine/Isoleucine hexoside	258.1333, 230.1393. 212.1287, 132.1021, 86.0976
4	328,14	98,2	-0,7	Phenylalanine hexoside	310.1272, 292.1151, 264.1202, 166.0836, 132.0785, 120.0783
5	474,23	98,9	-0,3	Ephedrine derivative 1 (+ hexosyl + deoxyhexosyl)	312.1832, 166.1219, 121.0650
6	188,07	99,2	1,3	Indoleacrylic acid	143.0722, 118.0649, 105.0334, 91.0555
7	206,04	99,8	0,1	Hydroxykynurenic acid	160.0389, 132.0441
8	235,11	96,1	-0,2	Unknown	146.0599, 128.0497
9	190,05	99,6	0,8	Kynurenic acid	144.0443, 116.0498, 89.0398
10	180,102	99,47	-0,08	Unknown	162.0906, 138.0913, 134.0956, 120.0804, 112.0761, 69.0342,
11	180,102	98,78	-0,15	Unknown	162.0915, 138.0913, 134.0958, 120.0816, 112.0750, 69.0340
12	516,24	97,9	1,4	Ephedrine derivative 2	354.1897, 166.1213, 121.0647
13	275,15	98,3	1,9	Unknown	137.0579, 112.0868, 70.0662
14	724,32	98,2	0	Ephedrine derivative 3	578.2604, 565.1553, 312.1822, 267.0855, 166.1234, 121.0646
15	565,15	99,2	0	Isoschaftoside	511.1178, 409.0920, 391.0830, 379.0787, 325.0711, 307.0599, 295.0611
16	750,33	97,9	-0,3	Ephedrine derivative 4	604.2745, 312.1797, 166.1222, 131.0489
17	566,43	96,9	-1,9	Unknown	548.4164, 435.3297
18	566,43	96,9	-1,9	Unknown	548.4164, 435.3297, 322.2475, 209.1619, 114.0908
19	625,18	99	0,6	Isorhamnetin *O*-hexoside-*O*-deoxyhexoside 1	317.0652, 85.0298
20	625,18	99,2	-0,5	Isorhamnetin *O*-hexoside-*O*-deoxyhexoside 2	317.0648, 85.0301
21	679,51	97,5	-1,4	Unknown (compound 18 + C6H11NO)	661.5002, 548.4152, 435.3350, 322.2484, 209.1635, 114.0922
22	433,11	98,4	0,2	Kaempferol 3-*O*-rhamnoside	287.0550, 85.0287, 71.0502
23	792,59	98,2	0,1	Unknown (compound 19 + C6H11NO)	774.5844, 661.4929, 548.4212, 435.3382, 322.2513, 209.1666, 114.0904
24	905,68	98,2	-0,3	Unknown (compound 20 + C6H11NO)	887.6590, 774.5781, 435.3322, 322.2532, 114.0913
25	1018,76	96,4	0,9	Unknown (compound 21 + C6H11NO)	
26	246,24	99,2	-1	Tetradecasphinganine	228.2288, 106.0864, 102.0907, 88.0763, 70.0660, 57.0705
27	274,27	98,1	-0,6	Hexadecasphinganine	256.2630, 230.2483, 212.2376, 106.0864, 102.0915, 88.0763, 70.0660, 57.0710
28	318,3	98,7	-0,1	Phytosphingosine	300.2897, 274.2765, 256.2626, 212.2370, 102.0916, 88.0761, 70.0654, 57.0708
29	290,27	98,8	-0,2	Sphingolipid derivative 1	272.2589, 242.2482, 118.0866, 88.0764, 74.0609
30	288,25	98	0,3	Sphingolipid derivative 2	242.2490, 116.0694, 102.0540, 88.0771, 74.0623, 57.0707
31	230,25	98,6	-0,9	Unknown	212.2364, 62.0610, 58.0661
32	318,3	98,4	0,3	Sphingolipid derivative 3	300.2865, 282.2772, 264.2678, 60.0822
33	258,28	97,9	-0,1	Unknown	240.2682, 58.0661
34	339.2505*	98,9	0,3	9,10-Dihydroxystearic acid	281,2478
35	286,31	98,2	0,2	Deoxysphinganine	268.2988, 58.0659
36	317.2088*	97,9	-0,8	Hydroxyoctadecatrienoic acid	95.0870, 81.0705, 57.0338
37	522,36	90,8	-2,8	Unknown (choline derivative)	504.3427, 445.2716, 184.0726, 104.1068, 86.0968

38	340,28	98,25	0,04	Unknown	298.2741, 280.2630, 142.1575, 109.1014, 95.0859, 83.0869, 69.0707, 60.0453, 55.0554
39	353,27	96,48	-1,66	Unknown	97.1014, 90.0558
40	282,28	98,2	0,4	Oleamide	264.2665, 247.2380, 121.1007, 97.1015, 83.0862, 69.0706
41	538,52	98,01	-1,04	N-Palmitoylsphingosine	309.2784, 256.2629, 102.0916, 88.0762, 70.0663
42	381,3	98,72	-0,81	Unknown	172.9869, 142.1525, 114.9853

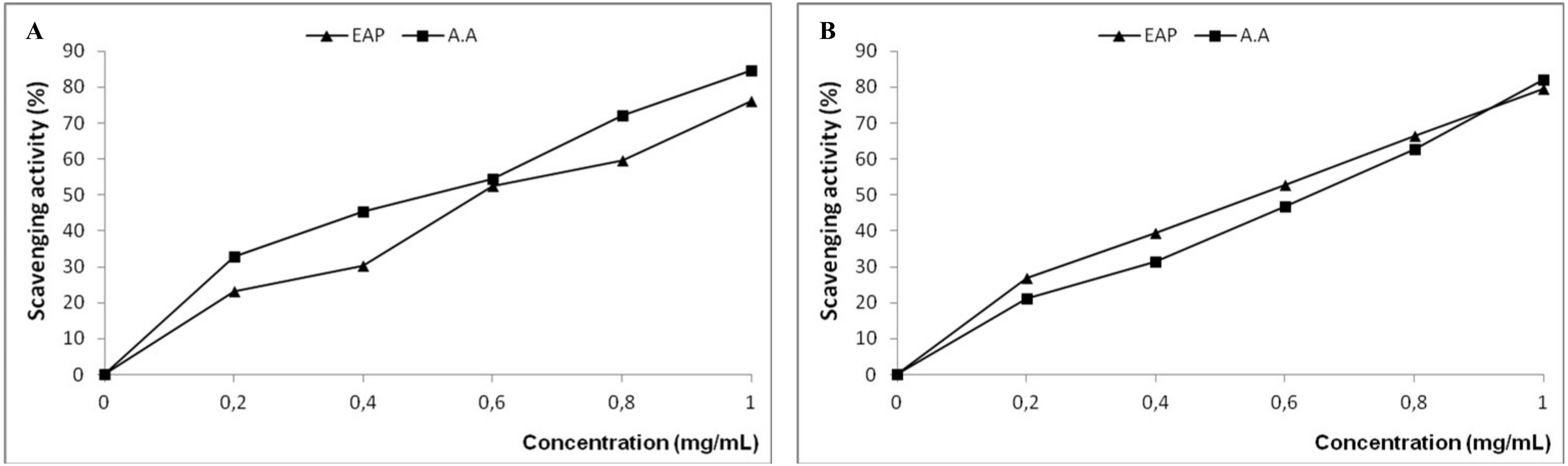

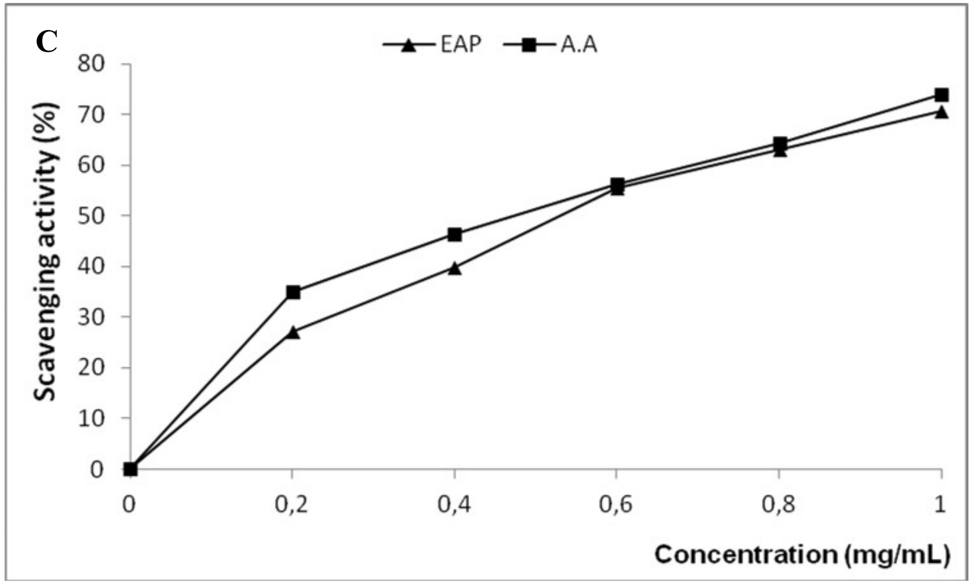

Figure S1. Antioxidant activities of EAP evaluated using DPPH (A), Superoxide (B) and Fe^{2+} chelating assays (C) in different concentration. Ascorbic Acid was used as a positive control. Values are means ± SD of three separate experiments EAP: *Ephedra alata* pulp. AA: Ascorbic Acid
